# Barriers and Enablers to Guideline-Directed Prescribing for Heart Failure with Reduced Ejection Fraction: A Multi-Stakeholder Qualitative Study

**DOI:** 10.5334/gh.1596

**Published:** 2026-10-30

**Authors:** Thin Mar Win, Audry Shan Yin Lee, Sameera Senanayake, Nicholas Graves, Sanjeewa Kularatna, Raymond Ching Chiew Wong, Seet Yoong Loh, Tan Yuyang, Huang Weiliang, Chow Weien, Jeanne Shan Yin Ong, Peck Kah Hua, Derek J. Hausenloy, Mark Yan-Yee Chan, Khung-Keong Yeo, Kheng Leng David Sim, Sungwon Yoon

**Affiliations:** 1 Health Services Research and Population Health, Duke-NUS Medical School, 8 College Road, Singapore; 2 Department of Cardiology, National Heart Centre, 5 Hospital Drive, Singapore; 3 National Heart Research Institute Singapore, National Heart Centre, 5 Hospital Drive, Singapore; 4 Australian Centre for Health Services Innovation, School of Public Health and Social Work, QUT Centre for Healthcare Transformation, Faculty of Health, Queensland University of Technology, 88 Musk Avenue, Kelvin Grove, QLD 4059, Australia; 5 Yong Loo Lin School of Medicine, National University Singapore, 10 Medical Dr, Singapore; 6 Department of Cardiology, National University Heart Centre, 5 Lower Kent Ridge Rd, Singapore; 7 Department of Cardiology, Tan Tock Seng Hospital, 11 Jalan Tan Tock Seng, Singapore; 8 Internal Medicine, Singapore General Hospital, Outram Road, Singapore; 9 Changi General Hospital, 2 Simei Street 3, Singapore; 10 Department of Medicine, Ng Teng Fong General Hospital, 1 Jurong East Street 21, Singapore; 11 Khoo Teck Puat Hospital, 90 Yishun Central, Singapore; 12 Cardiovascular & Metabolic Disorders Program, Duke-NUS Medical School, 8 College Road, Singapore; 13 The Hatter Cardiovascular Institute, University College London, 67 Chenies Mews, London, WC1E 6HX, United Kingdom; 14 Cardiovascular Disease National Collaborative Enterprise, 23 Rochester Park, #06-01, Singapore; 15 Duke-NUS Medical School, 8 College Road, Singapore

**Keywords:** Heart failure with reduced ejection fraction, Guideline-directed medical therapy, Quadruple therapy, Singapore

## Abstract

**Background:**

Heart failure with reduced ejection fraction remains a major cause of morbidity and mortality despite effective treatments. Guideline-directed medical therapy, commonly referred to as quadruple therapy, reduces mortality and hospitalisation; however, uptake and dose optimisation remain suboptimal across Asia, including Singapore. This study explores barriers and enablers influencing prescribing and titration practices in a multi-ethnic Asian setting.

**Methods:**

We conducted semi-structured interviews with 38 healthcare providers from seven public healthcare institutions in Singapore, including cardiologists, clinicians from relevant subspecialties, nurses, and pharmacists. The study was informed by the Cabana framework, and data were analysed using an inductive-deductive approach.

**Results:**

Guideline awareness was generally high, but confidence in initiating and up-titrating therapy varied. Attitudinal barriers included perceived gaps between clinical trial evidence and the realities of managing diverse patient populations, safety concerns in multi-morbid patients, clinical inertia, challenges related to professional boundaries, and limited confidence among junior staff. Nonetheless, guidelines were perceived as enablers, providing a shared framework that promotes standardised care. Prescribing behaviour was shaped by patient-related factors including financial constraints, limited health literacy, cultural preferences for traditional medicines, and treatment outcomes. Organisational barriers included limited access to specialist follow-up, rotating inpatient teams, formulary gaps in primary care, and fragmented medical records. Multidisciplinary nurse- or pharmacist-led titration clinics with specialist support were a key institutional enabler.

**Conclusions:**

To narrow the guideline-practice gap, targeted toolkits and specialist support are needed to address concerns and build staff confidence. A culture of reviewing quadruple therapy prescription at discharge and during follow-up should be supported. Prescribing patterns should be monitored and reflected in real-time, and patient adherence strengthened through dedicated heart failure nurses or case managers. Telehealth or primary care-based services, increased government support for healthcare information integration, and increased financial support for patients are necessary.

**Lay Summary:**

- Heart failure with reduced ejection fraction is a serious medical condition that can lead to frequent hospital admissions and premature death. Clinical guidelines strongly recommend the use of four classes of evidence-based medicines, commonly known as quadruple therapy, because they can significantly improve survival and reduce hospitalisation. Despite guideline endorsement, real-world uptake and dose optimisation of quadruple therapy remains suboptimal globally.
- Studies on implementation barriers in adhering to the guideline recommendations for quadruple therapy have focused mainly on physicians. This study provides one of the first multi-institutional qualitative accounts of barriers and enablers in prescribing quadruple therapy or guideline-directed medical therapy (GDMT) for heart failure patients in Singapore, exploring the views of all three frontline healthcare professional groups—doctors, nurses, and pharmacists.
- We found that the prescription of quadruple therapy was negatively influenced by a perceived gap between clinical trial evidence and the realities of managing diverse patient populations—particularly older and multimorbid patients—alongside interprofessional relationships and limited confidence among junior staff. Health care providers’ prescribing behaviour was shaped by patient-related factors, including financial constraints, limited health literacy, and cultural preferences for traditional medicines.
- Limited specialist follow-up, fragmented health records, and formulary gaps in primary care hinder implementation, while positive patient outcomes and multidisciplinary care models act as enablers in adhering to guideline recommendations.
- The study adds context-specific implementation insights by identifying individual, organisational, and system-level factors. Specialist-supported nurse- or pharmacist-led services, primary care-based medication titration, clearer task-sharing pathways, improved decision tools, and better health information integration may enhance guideline implementation.

## Graphic Abstract

**Figure:**
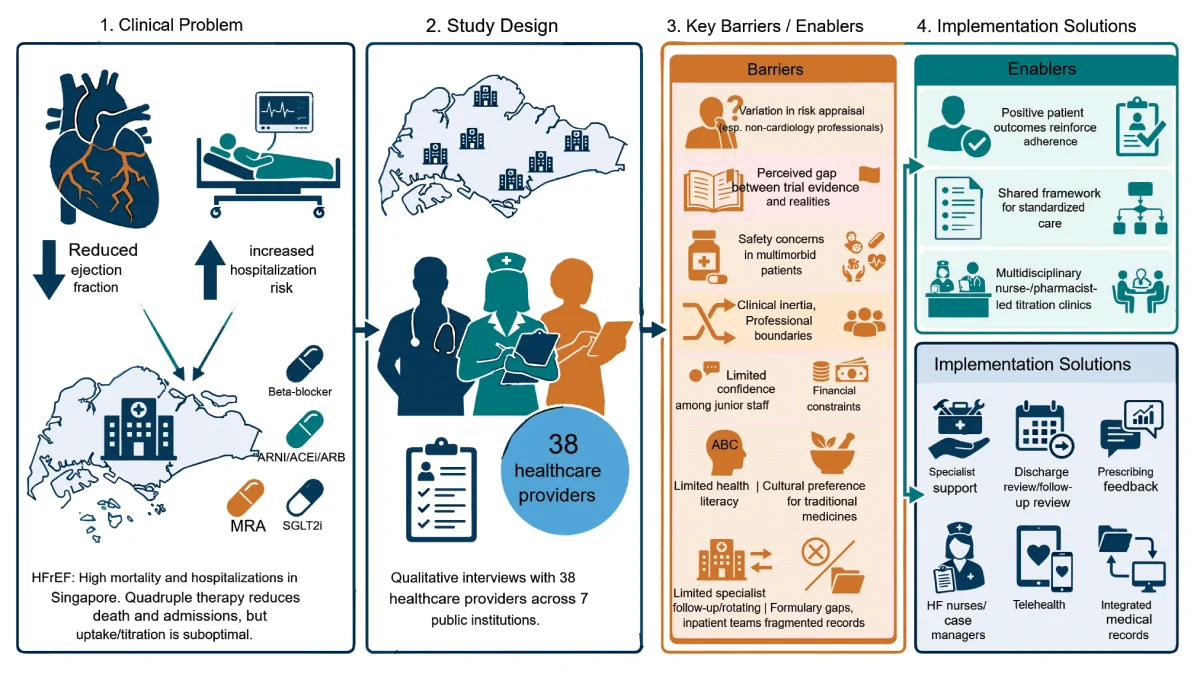
Graphical Abstract of ‘Barriers and enablers to guideline-directed prescribing for heart failure with reduced ejection fraction: A multi-stakeholder qualitative study.’

## Introduction

Heart failure affects more than 64 million people worldwide (1,2). Although advances in treatment have improved survival rates, the condition continues to impose substantial morbidity, mortality, and healthcare costs (3). Heart failure with reduced ejection fraction (HFrEF) in particular remains associated with high morbidity and mortality.

Guideline-directed medical therapy (GDMT) for HFrEF, commonly referred to as quadruple therapy, consists of four key drug classes: renin-angiotensin-aldosterone system inhibitors (RAASi), beta-blockers (BB), mineralocorticoid receptor antagonists (MRA), and sodium-glucose cotransporter-2 inhibitors (SGLT2i). Randomised controlled trials have shown that these therapies substantially reduce mortality and hospitalisation in patients with HFrEF (4,5,6,7,8,9). In a modelling study, GDMT has been estimated to reduce the combined risk of cardiovascular death or heart failure hospitalisation by as much as 72% (10). Globally, optimal implementation of quadruple therapy could prevent up to 1.2 million deaths in a single year, with the greatest potential impact being in Southeast Asia, the Eastern Mediterranean, Africa, and the Western Pacific (11).

Although the benefits of GDMT are well established and strongly endorsed by major cardiology guidelines (12,13), there is a substantial gap between recommended care and actual prescribing practice. Worldwide, only a minority of eligible patients with HFrEF receive all four recommended therapies and achieve target doses (11,14,15,16,17). Data from the ASIAN-HF registry from 2012–2015, which included more than 5,000 patients with HFrEF across several Asian regions, showed that GDMT was often under-prescribed or prescribed at suboptimal doses (18). Our previous quantitative study in Singapore also identified a substantial gap in GDMT implementation despite a high proportion of eligible patients (19). Low prescribing rates, inadequate dose optimisation, and high rates of discontinuation after initiation (18,20,21,22) highlight the urgent need for targeted strategies to improve initiation, titration, and adherence to guideline recommendations.

Given that suboptimal implementation of GDMT directly contributes to preventable morbidity and mortality, identifying the specific barriers and enablers across a range of healthcare professionals involved in heart failure services is a critical first step toward designing targeted implementation strategies that can increase GDMT uptake, support dose optimisation, and ultimately translate the proven efficacy of GDMT into improved population-level outcomes. While previous studies have explored the factors contributing to the treatment gap in quadruple therapy for patients with HFrEF from the perspectives of individual professional groups (23,24,25), a multidisciplinary perspective incorporating the experience of doctors, nurses, and pharmacists within the same healthcare system remains underexplored. Therefore, this study aims to explore the barriers and enablers influencing the prescription of quadruple therapy and to better understand the guideline-practice gap experienced by the multidisciplinary frontline healthcare workers in a multi-ethnic Asian healthcare context, to inform tailored implementation strategies.

## Methods

### Study design and participant recruitment

We conducted semi-structured, in-depth interviews with 38 healthcare providers (21 years to 65 years of age) from seven institutions under all three public healthcare clusters in Singapore. Additional information on the recruitment process and Singapore healthcare system is provided in the supplementary material. Participants included heart failure specialists, cardiologists, specialists from general medicine, geriatrics, and family medicine, as well as advanced practice nurses (APNs) and pharmacists involved in heart failure care. Potential participants were purposely identified by members of the study team at each institution based on their profession, job scopes, and years of experience in the workplace before being referred to the interviewer. The interviewer contacted them to confirm if they were willing to participate in the study. Of the 39 participants approached, one did not reply and was therefore excluded. The study was approved by the National University of Singapore Institutional Review Board (NUS-IRB-2025-339).

### Data collection

We developed a semi-structured interview guide informed by relevant existing literature and three main domains (knowledge, attitude, behaviour) from the Cabana Framework. The guide covered experience in managing HFrEF patients, awareness of and familiarity with quadruple therapy guidelines, attitudes towards guideline recommendations, and factors influencing guideline-directed prescribing. After pilot-testing, interviews were conducted online between July and November 2025 by a member of the study team who was trained in public health and qualitative research methods. The interviewer did not have any personal relationships with the participants. Verbal consent was obtained and recorded before each interview. All interviews were audio-recorded and conducted in English, lasting between 35–78 minutes. We used the same question guide for doctors, nurses, and pharmacists, but some different sections were included to capture the unique perspectives of each professional group.

### Data analysis

All interviews were transcribed verbatim. Data analysis involved both inductive and deductive analytic approaches (26,27). After familiarising the interview data, two members of the study team independently coded by naming or defining concepts. Through an iterative process, the data were classified into broad categories and subcategories that captured the key factors influencing guideline adherence. For example, provisional categories such as not wanting to add a new class of medication if the patient was already stable; if the patient was elderly; and if the explanation process on reasons for additional medication would take time were later merged into a category of clinical inertia. These inductively derived categories and subcategories were then examined in relation to three domains of the Cabana framework, to which they were mapped. The Cabana framework is a widely used conceptual model for identifying clinician-related factors that influence guideline adherence, as well as external and environmental influences such as patient acceptance, patient compliance, and organisational constraints (28). Regular discussions among the study team were held to refine and confirm categories and overarching dimensions. NVivo version 15 was used to support data organisation, coding, and analysis (29). The study was reported in accordance with the Consolidated criteria for reporting qualitative research (COREQ) (30).

## Results

The results are organised into four sections: participant characteristics, healthcare providers’ knowledge, healthcare providers’ attitudes, and healthcare providers’ behaviours. Within each section, we present the barriers and enablers identified. Figure 1 shows how the Cabana framework was applied to our findings.

**Figure 1:**
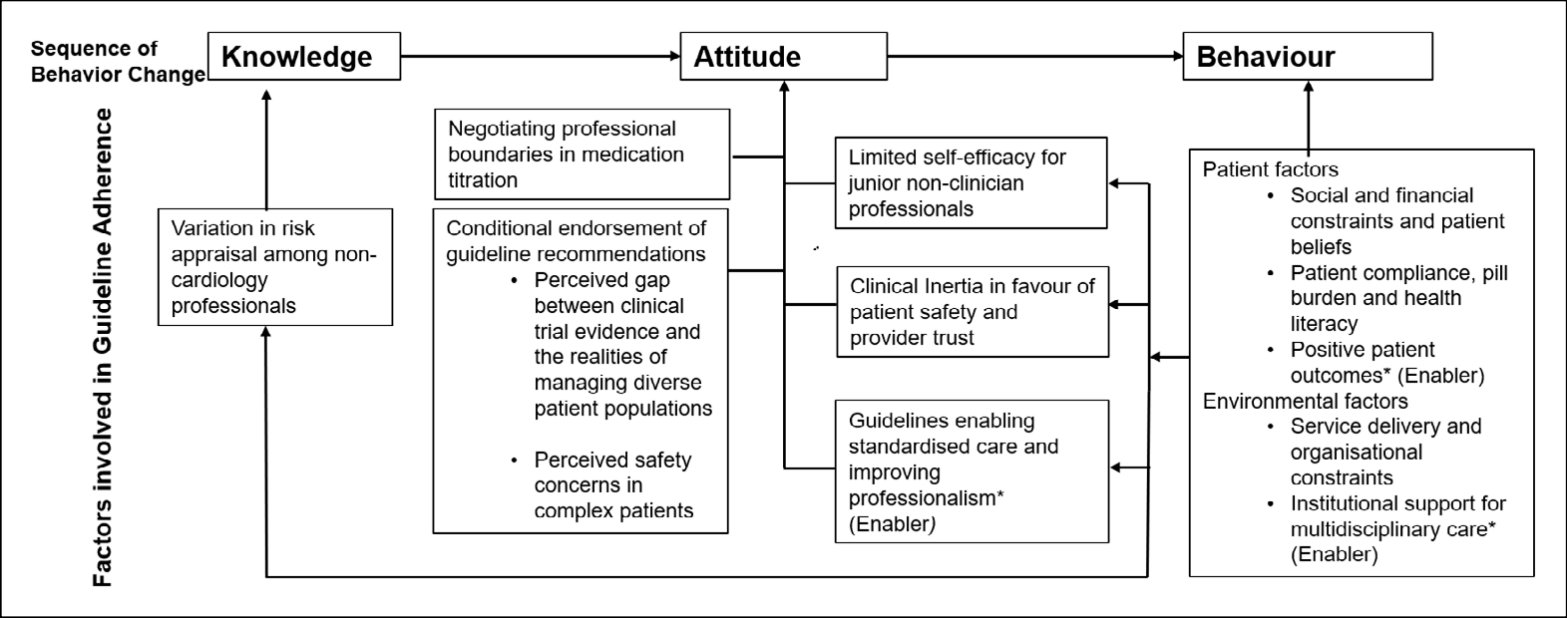
Cabana Framework applied to our study findings.

### Participant characteristics

Participant characteristics (n = 38) are presented in Table 1. All participants had a minimum of three years of clinical experience and more than one year of experience in prescribing or contributing to prescribing decisions related to GDMT. All pharmacists had completed collaborative prescribing training for heart failure care. All nurses were cardiology APNs involved in heart failure services, although two did not hold prescribing rights. Data saturation was reached at 35 interviews, and 3 interviews were additionally conducted beyond data saturation to ensure that point of information redundancy was achieved. Recurring patterns were evident within and across all three groups.

**Table 1:** Characteristics of participants (n = 38).


PARTICIPANT CHARACTERISTICS	NUMBER (%)

**Gender**	

Male	11 (28.9%)

Female	27 (71.1%)

**Types of health care providers**	

Doctors	18 (47.4%)

Advanced practice nurses	11 (28.9%)

Pharmacists	9 (23.7%)

**Clinical experience**	

>3 years	9 (23.7%)

>10 years	21 (55.3%)

>20 years	8 (21.0%)

**Experience in heart failure services**	

>1 year	6 (15.8%)

>3 years	7 (18.4%)

>5 years	25 (65.8%)

**Speciality (for doctors)**	

Heart failure specialists	9 (50%)

General cardiologists	3 (16.7%)

Internal medicine	2 (11.1%)

Geriatricians	2 (11.1%)

Family medicine	2 (11.1%)


### Healthcare providers’ knowledge

#### Variation in risk appraisal

All participants reported awareness of international and local heart failure guidelines, including those from Europe, America, and Singapore (31). This awareness was acquired through conferences, continuing medical education sessions, journal reading, and interactions with cardiologists. Most participants indicated that they aimed to follow either the European or American guidelines in routine clinical practice wherever possible. One participant from primary care reported following the Singapore heart failure guideline.

‘I follow the Singapore heart failure guideline. We should probably follow what [the] Ministry of Health (MOH) suggests.’ *(Clinician 4)*

Some non-cardiology doctors reported improved familiarity with the guidelines following educational sessions led by heart failure specialists on recent developments in quadruple therapy for heart failure.

‘I think everybody is familiar with quadruple therapy, as all the cardiology speakers talked about it. It has been one or two years already. So, I think there is no gap in knowledge between cardio and non-cardio.’ *(Clinician 8)*

However, heart failure specialists emphasised that awareness alone was insufficient, highlighting the need to strengthen confidence in applying quadruple therapy guidelines. They noted that some clinicians had knowledge gaps or felt uncomfortable initiating, up-titrating, or maintaining these medications, especially when blood pressure or renal function was a concern.

‘Some patients are seeing other physicians who might not be so well versed in GDMT. So, they tend to be more worried about low blood pressure. Sometimes the medications are stopped without a very good reason. We took a long time to up-titrate the medication…’ *(Clinician 13)*

‘Sometimes, we assume that everyone knows what GDMT is and is comfortable to initiate or up-titrate them, but in reality, not everyone may be similarly familiar with four classes or comfortable using all of them.’ *(Clinician 6)*

Some non-cardiology physicians also stated differences in the pace and threshold of medication up-titration in HFrEF management compared with cardiologists, noting a more cautious approach among non-cardiologists.

‘I feel their threshold, and their pace of titration, is very different from us. Cardiologists can be rather brave and aggressive, but for us, we tend to do [it] a bit slow.’ *(Clinician 9)*

### Healthcare providers’ attitudes

#### Conditional endorsement of guideline recommendations

##### Perceived gap between clinical trial evidence and the realities of managing diverse patient populations

Some participants felt that current guidelines were based on clinical trial populations that differed demographically from their local patients and often did not adequately represent very elderly individuals, particularly those aged over 85 years. As a result, some were hesitant to apply these guidelines aggressively in practice. This pattern was consistent across all professional groups.

‘Trials include a relatively younger cohort of patients. Not many of them have an aged population that you can extrapolate the evidence base. European guidelines do not fit Asian patients. The patients that I look after are half the size of the average European.’ *(Clinician 3)*

‘…in how aggressive we are to be managing the patient, especially when there might be a different population type compared to our local population. Their patients may be bigger, can tolerate higher doses. But compared to Asians who are smaller, frailer, how do we titrate accordingly?’ *(Nurse 1)*

‘I think when the guidelines first come out, we always do journal critique. And the lack of Asian representation is always a point raised.’ *(Pharmacist 6)*

##### Perceived safety concerns in complex patients

Although participants generally trusted the guidelines when treating younger patients with fewer comorbidities, they expressed reservations about their applicability to patients with more complex clinical profiles. Their main concerns centred on deterioration in renal function and adverse effects, especially hypotension and an increased risk of falls.

‘You try to maximize the class of each medication; sometimes it is not realistic because of the blood pressure and the effect on the kidneys.’ *(Clinician 1)*

‘Sometimes because of their [patient] renal function, we cannot start the medicine for them at all. That is one of the limiting factors. The other thing is low blood pressure. Then we are very fearful that they will fall.’ *(Nurse 1)*

Even when GDMT had been recommended by a cardiologist, one non-cardiology participant expressed hesitation about prescribing it because of concerns about patient safety.

‘If a cardiologist gives a recommendation to start certain things on, what we do is we also assess suitability again, mainly because the elderly with multiple comorbidities may or may not be so suitable. If blood pressure is a bit borderline, it is difficult to start.’ *(Clinician 18)*

Some non-cardiology participants noted that many hospitalised patients were either not suitable candidates for initiation of quadruple therapy or required temporary withholding of treatment because of acute clinical issues.

‘The problem with our patients is multimorbidity. Some have HFrEF and a new diagnosis of a malignancy during the acute hospitalization. Then, the GDMT becomes second thought. They may also have acute infection leading to hospitalisation, and SGLT2 may not be appropriate. Our cases are not clean cut like the patients seen by cardiologists.’ *(Clinician 8)*

#### Clinical inertia in favour of patient safety and provider trust

Clinical inertia refers to the failure to initiate or intensify therapy in patients who have not achieved evidence-based targets of care (32). Although participants often described hesitancy around initiating or up-titrating quadruple therapy in elderly patients, those with renal impairment or low blood pressure, and individuals with poor adherence, six participants explicitly stated that they preferred not to alter treatment in clinically stable patients to preserve the status quo. This was framed as prioritising patient safety, comfort, and trust. Participants also linked clinical inertia to time pressures, particularly a lack of time to explain medication changes and potential adverse effects.

‘Actually, when you introduce a new drug or increase the dose, and then the patient has a side effect, you’ve lost the trust of the patient and the family. So, if the blood pressure is good enough and they’re not being admitted to the hospital and stable, you have to ask yourself: is that a patient that does need to be pushed? It is called patient safety.’ *(Clinician 3)*

‘I suppose it is inertia on the medical team and inertia on a patient; both play. For [the] medical team, it is due to the challenges of too little time for each patient. So, if the patient seems well on [the] surface, you may not want to increase the medicine and change things and explain.’ *(Clinician 6)*

Clinical inertia was consistent among all professional groups.

‘Our end goal is to reduce readmission, which includes not just due to fluid overload but also adverse reaction from the GDMT. Given the fragility of the patient population that we are handling, sometimes I do agree with not rocking the boat and [to] just keep on to whatever GDMT that we have started and [the] patient is tolerat[ing]. If that [is] achieved, the goal of getting patients out of the hospital is achieved already.’ *(Nurse 7)*

#### Limited self-efficacy for junior non-clinician professionals

Some pharmacists and nurses reported that limited experience during their junior years in heart failure services contributed to delays in initiating and up-titrating GDMT. Over time, however, mentorship from senior staff and heart failure specialists helped build their confidence.

‘We don’t know when it is a good time to initiate the medicine because of the patient’s kidney function. Because of my lack of experience, I may wait and see a bit longer before I initiate medicine. So, confidence and experience are important.’ *(Nurse 9)*

‘The competency has improved along with the experience and the knowledge gained. Four or five years ago, I needed to consult with the cardiologist as I was not sure how to decide. Now, I am more confident.’ *(Pharmacist 9)*

#### Negotiating professional boundaries in medication titration

Effective delegation of medication up-titration to other healthcare providers depended on more than formal prescribing authority. Pharmacists and nurses with prescribing rights described that doctors’ referral notes were sometimes ambiguous. They tended to interpret this as a directive rather than a suggestion, narrowing their clinical judgement even though they had the authority to prescribe independently.

‘Most of the referral cases … Specialist will probably just say please recheck renal panel. Or they would say KIV up-titrate something, for example ARB or ACEI or KIV start X medication, that’s all they say.’ *(Pharmacist 8)*

A similar dynamic was evident among non-cardiology physicians. Some described holding back out of professional courtesy, recognising that the cardiologist retained a sense of ownership over the treatment plan. Their decision not to intervene was about respecting boundaries that had been informally established over time.

‘Cardiologists prefer to stick to their own appointments, and they do their own titration. So, out of courtesy, I don’t really interfere much with their plan.’ *(Clinician 10)*

#### Enabling standardized care and improving sense of professionalism

Despite these attitudinal barriers, some participants described guidelines as providing a shared framework for healthcare providers, thereby promoting more standardised and consistent clinical practice. Adherence to evidence-based guidelines was also seen as strengthening professionalism in care delivery.

‘The guideline is quite clear, written with all the available evidence. It is kind of [a] recipe book for everybody to follow so that we know that this is a standardized workflow for us.’ *(Nurse 6)*

‘Being a healthcare professional, following guidelines based on scientific evidence makes us ethical to patients and feel professional.’ *(Pharmacist 2)*

### Healthcare Providers’ Behaviours

Although appropriate knowledge and attitudes are important prerequisites, they alone are insufficient to ensure adherence to guideline recommendations. Participants reported that external influences—particularly patient-related and environmental factors—limited their ability to prescribe quadruple therapy. Social determinants of health and organisational constraints were identified in particular as key barriers to prescribing.

#### Patient factors

##### Social and financial constraints and patient beliefs

Quadruple therapy requires close post-initiation monitoring, including frequent laboratory tests, which can place a considerable burden on patients because of the need for repeated hospital visits. Participants reported that this was challenging for many patients. Among working-age adults, limited access to leave from work was a major barrier, whereas among older patients, reliance on family or caregiver support for transport to hospital or clinic was a key concern.

‘Some patients are less willing to come back so frequently because of work or distance or no caregiver.’ *(Clinician 2)*

Singapore operates on a shared responsibility model, and the cost of healthcare is borne by a combination of the government, the individual, and insurance. However, for patients who had stopped working because of their cardiac illness, transport costs—particularly taxi fares—were reported to impose a financial burden on them.

‘Because of heart failure, they find it hard to go to work. No work, no income. … [T]raveling here is an issue because they cannot take public transport, because they feel breathless. So seeing [a] doctor is actually too expensive for them.’ *(Nurse 2)*

These barriers were described as slowing the pace of medication up-titration. Although medical social workers can assist patients in applying for subsidies and financial support, some patients were either ineligible or unable to provide the required documentation. Participants also highlighted limitations in the use of MediSave, as conditions such as heart failure and atrial fibrillation are not included under Singapore’s Chronic Disease Management Programme (CDMP). The CDMP is a Ministry of Health initiative that allows patients to use MediSave for outpatient treatment of 23 common chronic conditions at accredited clinics (33).

‘Only certain chronic diseases such as high blood pressure, high cholesterol, diabetes, [and] ischemic heart disease are approved by MOH for patients to use the MediSave payment. I find it very peculiar why heart failure and atrial fibrillation are not included.’ *(Pharmacist 3)*

In addition, some patients declined prescribed treatment because of personal choice or a preference for traditional therapies. Collectively, these social, financial, and preference-related factors shaped clinicians’ management of HFrEF with quadruple therapy.

‘Some believe Chinese medicine more than Western medicine. I cannot do anything for that. I also got one patient last time, like 40-plus year old, EF 20%, then I want to start Entresto. He goes back [to] Google and then said: “Oh, Entresto potentially may cause dementia”. Then he refused to take Entresto.’ *(Clinician 17)*

##### Patient compliance, pill burden, and health literacy

Poor compliance, pill burden, and limited health literacy were among the most frequently cited challenges faced by healthcare providers in adhering to GDMT.

‘If they are not taking medication in the first place, then you might not want to give them so many medicines.’ *(Clinician 16)*

‘They resist to increasing the medication … With heart failure, why do I (patient) need to take so many medications? So, we have to re-educate them again. But sometimes even [when] we educate, they still don’t want [to].’ *(Nurse 4)*

‘Maybe half of my patients in the clinic are not paying much attention [to] the type of drugs, and they’re memorising medication by colours. They do not try to understand their disease condition. It is not directly related to education level. It is how much they pay attention [to] their health issue.’ *(Clinician 11)*

##### Positive patient outcomes as adherence reinforcement

Reports of positive patient outcomes—such as symptom improvement and reduced hospitalisations—were among the most cited enablers of adherence to quadruple therapy, particularly among healthcare providers who were able to follow patients over time.

‘What I usually find [to be] the most positive one is Entresto. Patients on all four and Entresto felt effort tolerance improvement.’ *(Pharmacist 4)*

‘We see that patients feel better. Less hospitalisation episode and their heart function improves.’ *(Clinician 2)*

#### Environmental factors

##### Service delivery and organisational constraints

Across all three public healthcare clusters in Singapore, timely follow-up with a cardiologist or heart failure specialist within two weeks of discharge was difficult to arrange. As a result, medication up-titration often relied on nurse-led and/or pharmacist-led heart failure clinics, where available. Although specialist appointments could be expedited for select patients based on clinical need, this was not feasible for all patients started on quadruple therapy.

‘After discharge, earliest visit with us may be about two to three months [later]. And then once a year if stable. They can always come back if they have some problem, but we have so many patients and to see them earlier is very difficult.’ *(Clinician 7)*

Some participants expressed frustration about the limited availability of certain medications in primary care. For example, zirconium or Resonium may not be available when patients are unable to tolerate alternative potassium binders. Similarly, eplerenone may be unavailable for patients who develop gynaecomastia while taking spironolactone.

Participants also described IT infrastructure as a barrier to coordinated care, as limited access to patient records across different healthcare settings made it difficult to manage patients receiving treatment in multiple locations. Clinicians emphasised the need for a single, longitudinal patient record that is accessible across clusters.

‘If I had one magic wand for Singapore … I’d make everybody on the same system. Private provider, community provider, hospital, all have the same set of notes so we can all read what is happening and have only one medication list.’ *(Clinician 3)*

Additionally, in one general hospital, the involvement of multiple providers and subspecialty-led rounding teams that rotated every three to four weeks contributed to variation in clinical decision-making, which in turn affected HFrEF management.

‘Every month with the on-board sub-specialization team with the different consultants, they may not know what we are doing. So, with all these factors, they’re bound to decrease uptake or not follow recommendation.’ *(Nurse 3)*

‘It’s a challenge because the different doctors are seeing the same patient in three weeks’ time, and their opinions can be different.’ *(Nurse 4)*

##### Institutional support for multidisciplinary care

A supportive multidisciplinary work environment was frequently identified as an important enabler of medication titration. This included access to nurse-led and pharmacist-led heart failure clinics for medication adjustment, together with heart failure specialist clinics running alongside these services on the same day.

‘We run clinics together because some of our pharmacists are not so confident in the physical examination. [I] must admit that my pharmacists are stronger in the field of pharmacology. Let’s say we notice that the result is very abnormal suddenly and [the] patient is not well … then we will just go over and consult with doctors.’ *(Nurse 2)*

‘I can see the patients after discharge in one month. If the allied health colleagues could help, time can be reduced to two weeks. Sometimes, they may remind me to up-titrate. This speeds up the number of patients that we can see.’ *(Clinician 12)*

## Discussion

This study explores the factors influencing adherence to guideline-directed prescribing among healthcare providers managing patients with heart failure across major public health institutions. Our findings revealed that despite high awareness of heart failure guidelines, uncertainty in applying guidelines to complex patients, clinical inertia, interprofessional role boundaries, and organisational limitations—such as follow-up capacity and formulary gaps—constrained optimal prescribing, while enablers such as multidisciplinary titration clinics and guidelines as a shared framework for standardised care supported guideline adherence. Our findings are consistent with previous international studies (23,24,34) and add important local context to understanding the guideline-practice gap.

Although participants generally demonstrated good awareness of quadruple therapy guidelines, confidence remained uneven among non-cardiology healthcare providers, contributing to hesitancy in up-titration and, at times, premature discontinuation. This variation in confidence across specialties is consistent with findings from previous work (35). Cross-specialty continuing medical education, decision-support tools, easy access to updated guidelines in routine practice, and clear communication channels among providers involved in heart failure care may help narrow this confidence gap.

Educational activities led by heart failure specialists, including practical guidance on managing renal function and blood pressure during quadruple therapy, may help address some of the commonly cited reasons for non-adherence (36). Nephrology and cardiology societies have also co-developed toolkits for the prevention and management of cardio-kidney complications. For example, the toolkit on optimising RAASi therapy and managing related complications includes practical, user-friendly infographics that can serve as point-of-care reference materials for nephrologists, cardiologists, and other healthcare professionals involved in multidisciplinary heart failure care (37).

In our study, participants described guidelines as providing a shared framework for standardised care and viewed adherence to recommendations as enhancing professionalism in practice. At the same time, they expressed concerns about medication-related adverse effects and about the applicability of existing guidelines to Asian patients, particularly those who are older, frail, and living with multiple comorbidities. One study involving older patients, with a median age of 73 years, found that early and rapid initiation of GDMT appeared both feasible and generally safe when blood pressure, renal function, and potassium levels were adequate, and treatment was implemented in carefully selected patients (38). However, direct evidence on the safety of GDMT in older Asian populations remains limited, and dedicated studies in these groups are still needed.

Importantly, clinical inertia was often justified in terms of patient safety and the preservation of trust between patients and healthcare providers. Some participants acknowledged preferring to maintain the status quo in clinically stable and asymptomatic patients rather than strictly adhering to guideline recommendations, and this inertia was sometimes reinforced by the time pressures of busy clinics. Strategies proposed in the literature to address clinical inertia include clinician audits to identify reasons for non-prescribing or failure to up-titrate, standardised visit-based checklists for GDMT, electronic alert systems in pre-discharge and ambulatory settings, and structured nurse-led and pharmacist-led follow-up clinics to support task-shifting and strengthen team-based care (39,40).

In our study, specialist involvement emerged as both an enabler and a constraint. Many nurses and pharmacists described cardiologist mentorship and collaborative support as empowering, particularly in managing complex patients, and as helping to build confidence in heart failure medication titration over time. However, this sense of empowerment was not universal. Some participants described working relationships in which the boundaries of their role felt implicitly defined by the specialist’s communication style and referral practices. These findings suggest that the value of specialist involvement depends not only on access to expertise, but on the quality of the interprofessional relationship through which that expertise is communicated. Approaches such as clear role delineation, supportive supervision, and structured referral pathways may help promote effective task-sharing while maintaining safety.

We also found that prescribing behaviours were shaped by patient-related factors and organisational constraints. Dedicated heart failure case managers or heart failure nurses may play an important role in improving patient adherence, health literacy, and self-management. Accordingly, these roles should focus primarily on heart failure care, including structured patient education, follow-up coordination, and telehealth support. Certain techniques such as ‘teach-back’, assessments of patients’ self-management knowledge, and escalation plans can be explored in the local context (41,42,43).

For many patients with heart failure, frequent follow-up during up-titration is intended to assess symptoms and arrange blood tests. In Singapore, family medicine specialists and community nurses already play important roles in hospital-at-home programmes. Leveraging these community-based resources for symptom monitoring and medication up-titration (44), together with telehealth-supported titration models (45,46), may reduce the need for hospital visits and lower travel-related costs for patients. Patients and healthcare providers in Singapore have reported valuing blood pressure telemonitoring and teleconsultation for their convenience and support for self-management, although challenges such as usability and technology-related anxiety remain (47). Further evaluation of telehealth for heart failure care is therefore warranted, even in a geographically small country such as Singapore. In addition, formulary limitations across polyclinics for medications that support adherence to quadruple therapy, such as potassium binders and alternative MRAs, should be addressed. Laboratory investigations needed for follow-up should also be made more accessible through polyclinics and home-based services. Finally, better integration of electronic health records should be prioritised to reduce duplication and miscommunication.

While our study focused on the perspectives of frontline healthcare professionals, several of the barriers we identified also reflect health system and policy-level issues. Addressing these barriers will likely require complementary policy and heath-system-level interventions, including enhanced medication subsidies; financial supports; inclusion of heart failure therapies in standard formularies across public primary care polyclinics and restructured hospitals to ensure affordability and accessibility; and integration of clinical information across care settings.

### Strengths and limitations

This study included all major tertiary healthcare institutions in Singapore and recruited a diverse group of healthcare professionals to provide a comprehensive, nationwide, system-level perspective. The use of combined inductive and deductive analysis, mapping to the Cabana framework, and adherence to the COREQ guideline all strengthen the methodological rigour of the study.

However, the study also has several limitations. Although interviews were conducted by a trained qualitative researcher with no prior relationship to participants, social desirability bias cannot be entirely discounted. Heart failure medication titration is a practice area with well-established guideline recommendations, and participants may have felt inclined to present their clinical practices in a favourable light, particularly when discussing adherence to evidence-based care. In addition, the findings are based on self-reported practices, which are subject to recall bias and may not fully reflect actual prescribing behaviour. The public hospital-based sample may also underrepresent the perspectives of healthcare professionals working in private sectors or public primary care clinics. We also acknowledge that our study focused exclusively on the perspectives of healthcare professionals and did not capture the voices of patients and caregivers, whose lived experiences with medication costs, adherence challenges, and health literacy are central to several of the barriers identified. In addition, the findings are specific to the Singapore healthcare context and may not be fully transferable to other settings, although many are consistent with international literature.

### Recommendations for practice

To narrow the guideline-practice gap, strategies should focus on increasing confidence among non-clinicians and non-cardiology staff with structured toolkits, including step-by-step guidance for managing perceived clinical concerns such as renal complications and hypotension, with sufficient cardiology support. GDMT optimisation should be the default plan at discharge and during outpatient follow-up, unless contraindicated. Consideration should be given to computerised clinical decision-support tools and through multi-disciplinary approaches. Access to real-time data reflecting quadruple therapy prescription rates may help address clinical inertia and encourage guideline adherence. Patient-centred adherence support delivered through dedicated heart failure case managers or heart failure nurses is important, and tools such as ‘teach-back’ or escalation strategies should be further explored. Rapid up-titration may have to be supported via different models of care that allow for home-based telehealth or primary care-based up-titration as opposed to tertiary hospital-based appointments. Increased government support may be required for national healthcare information integration, and consideration for strengthening financial support, enhanced medication subsidies, and inclusion of heart failure therapies in standard formularies across primary care settings and public hospitals. In addition, task-sharing models with allied health professionals are likely to be most effective when specialist input is collaborative and capacity-building rather than restrictive. Supportive supervision and clear communication pathways may help reduce ambiguity, strengthen interdisciplinary trust, and support timely optimisation of therapy while maintaining patient safety.

## Data Availability

The data are not publicly available due to ethical concerns. The data presented in this study will be available on reasonable request to the corresponding author.

## Abbreviations

ASIAN-HF: Asian Sudden Cardiac Death in Heart Failure Registry

APNs: Advanced practice nurses

BB: Beta-blockers

COREQ: Consolidated Criteria for Reporting Qualitative Research

CDMP: Chronic Disease Management Programme

GDMT: Guideline-directed medical therapy

HFrEF: Heart failure with reduced ejection fraction

KIV: Keep in View

MOH: Ministry of Health

MRA: Mineralocorticoid receptor antagonists

RAASi: Renin-angiotensin-aldosterone system inhibitors

SGLT2i: Sodium–glucose cotransporter-2 inhibitors
